# Changes in COVID-19 risk perceptions: methods of an internet survey conducted in six countries

**DOI:** 10.1186/s13104-021-05846-8

**Published:** 2021-11-25

**Authors:** Felicia Zhang, Shu-Fang Shih, Harapan Harapan, Yogambigai Rajamoorthy, Hao-Yuan Chang, Awnish Singh, Yihan Lu, Abram L. Wagner

**Affiliations:** 1 Department of Epidemiology, School of Public Health, University of Michigan, Ann Arbor, MI 48109 USA; 2 Department of Health Administration, College of Health Professions, Virginia Commonwealth University, Richmond, VA 23298 USA; 3 Medical Research Unit, School of Medicine, Universitas Syiah Kuala, Banda Aceh, 23111 Indonesia; 4 Tropical Disease Centre, School of Medicine, Universitas Syiah Kuala, Banda Aceh, 23111 Indonesia; 5 Department of Microbiology, School of Medicine, Universitas Syiah Kuala, Banda Aceh, 23111 Indonesia; 6 Department of Economics, Faculty of Accountancy and Management, Universiti Tunku Abdul Rahman, Sungai Long Campus, Jalan Sungai Long, Cheras, 43000 Kajang, Selangor Malaysia; 7 School of Nursing, College of Medicine, National Taiwan University, Taipei, 10051 Taiwan; 8 Department of Nursing, National Taiwan University Hospital, Taipei, 10051 Taiwan; 9 National Technical Advisory Group on Immunisation Secretariat, National Institute of Health and Family Welfare, New Delhi, 110067 India; 10 Key Laboratory of Public Health Safety (Ministry of Education), Fudan University School of Public Health, Shanghai, 200032 China

**Keywords:** COVID-19, Vaccine hesitancy, Risk perceptions, Behavioral/attitudinal changes

## Abstract

**Objectives:**

This study assessed changes in behaviors/attitudes related to the COVID-19. With the understanding that behaviors and vaccine decision-making could contribute to global spread of infectious diseases, this study collected several waves of internet-based surveys from individuals in the United States, mainland China, Taiwan, Malaysia, Indonesia, and India. The aims of this study were to (1) characterize the relationship between the epidemiology of disease and changes over time in risk perceptions, knowledge, and attitudes towards hygienic behaviors; (2) examine if risk perceptions affect acceptance of less-than-ideal vaccines; and (3) contrast adherence to public health recommendations across countries which have had different governmental responses to the outbreak.

**Data description:**

We conducted cross-sectional online surveys in six countries from March 2020 to April 2021. By the end of June 2021, there will be six waves of surveys for the United States and China, and four waves for the rest of countries. There are common sets of questions for all countries, however, some questions were adapted to reflect local situations and some questions were designed intentionally for specific countries to capture different COVID-19 mitigation actions. Participants were asked about their adherence towards countermeasures, risk perceptions, and acceptance of a hypothetical vaccine for COVID-19.

## Objective

The goal of this project is to understand the international dimensions of behavioral changes and vaccine decisions that members of the general population make regarding COVID-19. The six countries studied in this project have had diverse mitigation responses to COVID-19. As of March 22, 2020, at the start of this project, China had 81,397 cases; US 32,057; Malaysia 1306; Indonesia 514; India 376; and Taiwan 169 [1]. Their on-going responses reflected the epidemiological circumstances and have changed over time [2]. The on-going roll-out of the COVID-19 vaccine has also differed widely across the countries. For example, the COVID-19 vaccination rollout started January 13, 2021 in Indonesia, February 24, 2021, in Malaysia, and March 22, 2021, in Taiwan, with the rate of vaccination uptake varying widely across countries [3, 4].

The aims of this study were to characterize the relationship between the epidemiology of disease and the changes over time in risk perceptions, knowledge, and attitudes towards hygienic behaviors; examine if risk perceptions affect acceptance of less-than-ideal vaccines; and contrast adherence to public health recommendations across countries which have had different governmental responses to the outbreak.

## Data description

We used an opt-in internet-based sample. A survey research firm obtained potential participants from social media and online advertisements and collected contact information and basic demographic data. Basic information about these panelists is available online [5]. At each wave, the research firm sent a link to the survey to a subset of these panelists. The full sets of questionnaires by wave, along with Table A, which describes the sample size and dates of data collection, are publicly available at: 10.6084/m9.figshare.14792058.

The required sample size is around 800 for most countries for each wave. With an alpha of 0.05, a power of 80%, and a proportion of 50% (a statistically conservative estimate of what proportion of the population supports a given public health action), the margin of error will be 4%. This margin of error will allow us to assess substantial trends over time. We sampled more individuals in the US across six waves to do sub-analyses by race/ethnicity and to track changes in behaviors at a finer scale.

We used quota-based sampling, such that the distribution of individuals by age and gender in this population reflected that of the adult population in each country [6]. Subsequently, we created weights for each country using a raking procedure [7], which reflected the distribution of age, gender, and region within a country (and race/ethnicity in the US). These weights do not account for differential access to internet. The age ranges sampled was similar across countries. All adults ≥ 18 years were eligible, except for Taiwan, where adults ≥ 20 years were eligible.

Outcomes of interest include mask-wearing behavior, measured by the number of days an individual went outside the house in the past week and how often they wore a mask. Similar with how it was asked for previous pandemics [8], we asked individuals their perceived likelihood of acquiring SARS-CoV-2 in the next month and their perceived risk of death if infected. Vaccination intent was measured differently for waves 1–5 versus after. In waves 1–5, we embedded vaccination intent in an experiment, where we first randomized to receive different information about a vaccine. A COVID-19 vaccine was said to be either 95% or 50% effective and to have a 5% or 20% risk of side effects like fever. These numbers were based off the range of plausible values when the survey was first created March 2020, with the effectiveness bounds based on influenza and measles vaccines [9, 10]. In addition to the international samples, this survey experiment was also embedded within a study in Detroit, MI, and in another study in Shanghai, China [11, 12].

Starting in wave 6, we asked individuals vaccination questions based off draft questions from the National Institutes of Health (NIH) Community Engagement Alliance Against COVID-19 Disparities (CEAL).

Vaccine hesitancy was measured through a 10-item scale, which was developed by the World Health Organization (WHO) Strategic Advisory Group on Immunization (SAGE) Vaccine Hesitancy Working Group for parental attitudes towards pediatric vaccines [13]. We found our adaptation, the adult Vaccine Hesitancy Scale (aVHS), to be highly related to influenza and COVID-19 vaccination behaviors [14]. Table 1 provides an overview of the datasets.

**Table 1:** Overview of data files/data sets

Label	Name of data file/data set	File types (file extension)	Data repository and identifier (DOI or accession number)
Data files	COVID-19 vaccine hesitancy surveys	SAS dataset (.sas7bdat)	ICPSR (10.3886/E130422V2) [15]

## Limitations

This survey used Internet-based samples, which allowed us to rapidly collect information and to avoid person-to-person contact. However, internet samples may have inherent biases. There is sampling bias in that individuals who participate need to have access to the internet, so individuals of lower socioeconomic status will be less likely to participate. With the exception of the vaccine questions, we tried not to change questions across surveys to maintain comparability. An education variable was only asked starting in wave 6. We pre-tested the questionnaires in all countries to make sure the language used is at a 6th grade level and is clear without ambiguity or confusion. Additionally, individuals may answer rapidly with little thought. We eliminated individuals who took less than 180 s on the survey, and we required individuals to answer each question for each survey, except for wave 1 and for all waves in Taiwan due to the requirements by the IRB.

## Data Availability

All data available at ICPSR at 10.3886/E130422V2 [15]. See Table 1 for details.

## Abbreviations

aVHS: Adult Vaccine Hesitancy Scale
CEAL: Community Engagement Alliance Against COVID-19 Disparities
NIH: National Institutes of Health
SAGE: Strategic Advisory Group on Immunization
WHO: World Health Organization
